# Novel ways to monitor immunosuppression in pediatric kidney transplant recipients—underlying concepts and emerging data

**DOI:** 10.1186/s40348-021-00118-8

**Published:** 2021-07-26

**Authors:** Thurid Ahlenstiel-Grunow, Lars Pape

**Affiliations:** Department of Pediatrics II, University Hospital of Essen, University of Essen-Duisburg, Hufelandstraße 55, 45147 Essen, Germany

**Keywords:** Biomarkers, Immune monitoring, Virus-specific T cells, Kidney transplantation, Pediatric transplantation, Immunosuppression, Viral infections

## Abstract

After pediatric kidney transplantation, immunosuppressive therapy is given to avoid acute and chronic rejections. However, the immunosuppression causes an increased risk of severe viral complications and bacterial infections and is associated with serious side effects. It is therefore crucial to achieve the optimal individual balance between over- and under-immunosuppression and thereby avoid unnecessary exposure to immunosuppressive drugs. In routine use, steering of immunosuppressants is performed primarily by monitoring of trough levels that mirror pharmacokinetics (although not, however, pharmacodynamics). Other diagnostic and prognostic markers to assess the individual intensity of immunosuppression are missing. Potential methods to determine immune function and grade of immunosuppression, such as analysis of the torque teno virus (TTV) load, QuantiFERON Monitor®, and ImmuKnow® as well as virus-specific T cells (Tvis), are currently being evaluated. In some studies TTV load, QuantiFERON Monitor® and ImmuKnow® were associated with the risk for post-transplant rejections and infections, but randomized controlled trials after pediatric kidney transplantation are not available. Post-transplant monitoring of Tvis levels seem to be promising because Tvis control virus replication and have been shown to correlate with virus-specific as well as general cellular immune defense, which represents the individual’s susceptibility to infections. Additional Tvis-monitoring provides an innovative opportunity to personalize the antiviral management and the dosing of the immunosuppressive therapy after pediatric kidney transplantation to avoid unnecessary therapeutic interventions and identify over-immunosuppression.

## Introduction

For many years, kidney transplantation has been the preferred treatment for pediatric patients with end-stage renal disease. To avoid acute and chronic graft rejections, lifelong immunosuppressive therapy is necessary, but this is associated with the risk of serious bacterial and viral complications. Moreover, immunosuppressive medication has side effects which include nephrotoxicity, arterial hypertension, anemia, leucopenia, new-onset diabetes mellitus, dyslipidemia, hypertrichosis, bone mineral disorders, growth impairment, malignancies, and delayed sexual maturation [1–6]. It is therefore crucial to achieve the optimal individual balance between over- and under-immunosuppression and thereby avoid unnecessary exposure to immunosuppressive drugs.

The immunosuppressive treatment impairs the individual cellular immune response resulting in an elevated incidence of severe viral complications, notably after pediatric transplantation. Post-transplant primary infections or reactivations, especially by cytomegalovirus (CMV), BK polyomavirus (BKPyV), or Epstein-Barr virus (EBV) are associated with increased morbidity, mortality, and graft failure, for example from CMV disease [7, 8], BKPyV-associated nephropathy [9, 10], and EBV-associated post-transplant lymphoproliferative disease [11]. The outcome of post-transplant viral infections is individually different, but prognostic markers are missing. Virus DNA and serology are the diagnostic standards used to guide immunosuppressive and antiviral therapy in the case of primary virus infections or reactivations, but they are insufficient to precisely predict the individual risk of viral complications. An antiviral prophylaxis or preemptive therapy is often recommended, especially for CMV, but antiviral medication should be restricted to patients with insufficient immune defense because of the high costs and severe side effects [12]. If antiviral drugs are not available, a preemptive reduction of immunosuppressive therapy is often performed in case of post-transplant DNAemia to avoid viral complications, especially for BKPyV [9] or for EBV [13, 14], but on the other hand, it is associated with an increased risk of under-immunosuppression and rejections. Accordingly, in case of self-limiting DNAemia, the preemptive reduction of immunosuppressive therapy is not only unnecessary but also hazardous. Because of the lack of prognostic markers, it is actually difficult to limit therapeutic interventions to patients with increased risk of viral complications like CMV disease, BKPyV-associated nephropathy, and post-transplant lymphoproliferative disease. The necessity of antiviral prophylaxis, the timing of antiviral therapy, and the optimal dosing of immunosuppressive therapy therefore remain a subject for debate, inviting exploration of new biomarkers with the aim to characterize over-immunosuppression and preemptively identify patients with the risk of viral complications and need for therapeutic intervention.

In routine use, diagnostic and prognostic markers to assess the individual intensity of immunosuppression are missing. Monitoring of immunosuppressive treatment is most often performed using pharmacokinetic (PK) monitoring by measuring trough levels of immunosuppressants. Unfortunately, the lack of validated PK exposure targets and the difficulty in extrapolating the pharmacodynamics (PD) from current exposure levels are a major limitation in the routine application of PK monitoring. Actually, no direct assays that measure the pharmacodynamic effect of immunosuppressants are available for routine use.

Accordingly, the search for appropriate markers of the individual strength of the immune response is crucial to achieve the aim of a personalized immunosuppressive therapy. Currently, ImmuKnow®, QuantiFERON Monitor®, and monitoring of the torque teno virus (TTV) load or virus-specific T cells (Tvis) are evaluated to characterize the individual immune function and the intensity of immunosuppression. Representing virus-specific as well as general cellular immune response, the analysis of Tvis level seems to be one of the most promising methods to steer antiviral and immunosuppressive therapy after pediatric kidney transplantation (Table 1).

**Table 1:** Methods for immunemonitoring

Method	Studies
Cytokines, chemokines	[15, 16]
ImmunoKnow-Assay	[17–21]
TorqueTenoVirus	[16, 22–29]
Virus-specific T cells	[30–34]

## Innovative non-cellular biomarkers

In the last few years, multiple methods to measure the grade of immunosuppression and thus to enable effect-related immune monitoring have been assessed. Cytokines and chemokines, especially IL-6 that can be routinely measured in most hospitals, showed some correlation with clinical events after pediatric kidney transplantation, but they seemed to be insufficient for immune monitoring and failed to predict future infectious events [35]. Different markers in the urine, which include proteins such as perforin, granzyme B, CXCL9, CXCL10, CXCR3 or CD3E, or mRNAs, as well as markers in the blood, for example, donor-specific antibody (DSA) functionality, TRIB1, FOXP3, kSORT, miR-142-5p, T cell subgroups, IFNy-ELISpot, B cell-related genes, and others, have been tested for their relation to immunological events such as acute and chronic rejection or operational tolerance. However, none of these has been validated in independent cohorts [15].

Functional assays to determine immune function seem to be a promising tool to optimize the dosing of immunosuppressants after solid organ transplantation. ImmuKnow® (Cylex, Columbia, MD, USA) is a commercially available assay measuring the amount of intracellular adenosine triphosphate (ATP) produced by CD4 T cells from whole blood after stimulation with phytohemagglutinin. Some observational trials observed low ATP levels in association with infections and high ATP levels in case of rejections after kidney transplantation [17] whereas other studies did not find any predictive value of ImmuKnow® assay for rejection or infection after kidney transplantation, especially after T cell depleting induction therapy [18]. In a randomized controlled trial after liver transplantation, the adjustment of the immunosuppressive therapy was based on ATP concentration of ImmuKnow® assay and resulted in less bacterial and fungal infections without influence to rejection rate [19]. However, randomized controlled trials after kidney transplantation are missing and the ImmuKnow® data are inconsistent and a meta-analysis of six studies did not support the use of the ImmuKnow® assay to predict the risk of infections and acute rejections after renal transplantation [20]. Another commercially available functional assay is the non-pathogen-specific QuantiFERON Monitor® test (Qiagen) detecting global cell-mediated immune response by plasma interferon-gamma (IFNy) levels after stimulation of the whole blood with a combination of antigens. An observational cohort study after solid organ transplantation using the QuantiFERON Monitor® showed significant lower IFNy levels in case of post-transplant infections [21], but interventional as well as pediatric data are lacking. Further studies are necessary to define cut-off levels and to analyze the benefit of intervention based on QuantiFERON Monitor® test. Neither ImmuKnow® nor QuantiFERON Monitor® reached clinical practice to date.

The torque teno virus (TTV) load was thought to serve as a possible endogenous marker of the immune function. TTV is a non-pathogenic, ubiquitous, circular single-strand DNA virus which mirrors the net state of immunosuppression [22]. TTV load increases under immunosuppressive therapy, and replication is inversely correlated with the number and function of T cells [22–24]. Several studies reported an association of TTV load with the post-transplant presence of infections or rejections [16, 25–27], and recently, an observational trial confirmed that high TTV load is related to an increased risk of post-transplant infections and decreased risk of rejections after kidney transplantation [28]. However, in liver-transplanted children, it was reported that TTV replication is influenced not only by immune status but also by viral coinfection resulting in lower TTV load in case of hepatitis E virus infection [29]. Many studies showed a poor quality with a low level of evidence [22], the results varied, and pediatric data after kidney transplantation are missing. To assess the impact of TTV load as a surrogate marker after kidney transplantation, the measurement technique should be standardized and interfering factors as age, immunosuppressive regimen, and coinfection with other virus types have to be characterized [22]. Until now, there have been no randomized controlled interventional trials using TTV viral load for steering of immunosuppression and confirm an additional benefit of post-transplant TTV monitoring. Future studies will show whether the measurement of TTV replication will become an additional biomarker to steer immunosuppressive therapy.

### Virus-specific T cells as a promising biomarker

Virus-specific T cells (Tvis) have been shown to play a key role in the control of virus replication [36]. Virus-specific CD4-positive T cells (CD4 Tvis) detect viral epitopes which are presented on major histocompatibility complex (MHC) class II molecules on antigen-presenting cells such as B lymphocytes, dendritic cells, and macrophages, and CD8-positive T cells (CD8 Tvis) locate and destroy virus-infected cells which present viral antigens by MHC class I molecules. Antigen-specific CD4 and CD8 T cells have been identified in most studies after stimulation with pooled peptides derived from virus lysate representing a broad answer against all viral surface antigens.

Also in children, it has been shown that the number of Tvis is associated with the risk of virus diseases [30–33]. Regarding the high incidence of post-transplant viral complications (especially in children), prophylaxis, diagnosis, and treatment of viral infections may be improved by the inclusion of Tvis-analysis in routine monitoring after kidney transplantation [36, 37]. Besides, there is increasing evidence that Tvis mirror not only the virus-specific but also the general cellular immune defense. Thus, Tvis may serve as a marker to identify over-immunosuppression [36–38].

A number of different assays are currently available for the detection of the cellular response to viral antigens [36], the main ones being the enzyme-linked immunospot (ELISpot) assay, the enzyme-linked immunosorbent assay (ELISA), intracellular cytokine staining followed by fluorescence-activated cell sorting (FACS) analysis, and MHC multimer staining. ELISpot, ELISA, and FACS assays are based on virus-specific in vitro stimulation of T cells inducing activation markers. Stimulation is performed by antigens such as virus-infected cell lysates, virus particles, proteins, or peptides. The easiest methodology is the ELISA, where cytokines such as IFNy can be measured in the supernatant of stimulated cells, and the ELISpot assay where IFNy is locally captured in microtiter plates. The disadvantage of these methods is that they do not allow a subclassification of stimulated cells, i.e., in CD4- and CD8-positive cells. The FACS analysis using intracellular cytokine staining followed by flow cytometry overcomes this drawback and allows a complete sub-characterization of Tvis, but comes at the price of a longer and more difficult methodology. In contrast, MHC multimer staining is rapid and independent of stimulation but has the disadvantage that special MHC/peptide complexes have to be manufactured for each antigen and MHC allele so that this test is expensive and cumbersome, making it unfeasible for use in routine care.

### Steering of immunosuppressive therapy by virus-specific T cells

Post-transplant monitoring has shown that the Tvis-levels fluctuated depending on the intensity of immunosuppression in pediatric kidney recipients. During the initial post-transplant period—at the time of intensified immunosuppressive therapy—the number of Tvis was decreased and showed an increase after reduction of the immunosuppressive drugs (own data, not published). Interestingly, the risk of viremia, such as EBV, correlated with the number of CD4 Tvis, not only against EBV but also against other virus types as CMV and adenovirus (ADV) (own data, not published). Accordingly, it is hypothesized that Tvis represent not only the virus-specific but also the general cellular immune defense and thereby correlates with the individual susceptibility to infections. It is also speculated that a high number of Tvis could also represent underimmunosuppression and might therefore be associated with a higher risk for immunological events such as acute and chronic cellular and humoral infections. Serving as a marker of over- or under-immunosuppression, additional post-transplant monitoring of Tvis levels might therefore optimize dosing of immunosuppressive drugs compared to blood level monitoring alone. On this basis, we conducted the investigator-initiated, multicenter IVIST trial, which is the first randomized, controlled trial to consider the benefit of additional steering of immunosuppressive drugs by Tvis levels using intracellular cytokine staining followed by FACS analysis [34, 38]. Sixty-four pediatric kidney recipients were randomized 1:1 4 weeks after transplantation either to a control group (n=33) with classical trough level monitoring of immunosuppressants or to an intervention group (n=31) with additional steering by Tvis levels. Regarding high prevalence, even in childhood and long-term persistency after primary infection, CD4 Tvis against CMV, ADV, and herpes simplex virus (HSV) were selected as being most suitable for monitoring after pediatric kidney transplantation. Both study groups received the same immunosuppressive regimen consisting of cyclosporin A and everolimus, with the same target range of trough levels. The primary endpoint of the study was the estimated glomerular filtration rate (eGFR) 2 years after transplantation. Secondary endpoints were the number and severity of viral infections and the exposure to immunosuppressive drugs, as well as biopsy-proven acute rejections and safety. In terms of an effect-related drug monitoring, the study design of the IVIST trial aimed to realize the personalization of immunosuppressive management after transplantation. The primary analysis detected no difference in eGFR between the intervention and control group 2 years after transplantation [34]. Patients in the intervention group received significantly lower daily doses (mg/m^2^) of everolimus (0.8±0.3 vs. 1.2±0.5, p=0.004) and numerically lower doses of cyclosporin A (78.4±20.0 vs. 88.4±26.5, p=0.13) resulting in significantly lower trough levels (μg/L) of everolimus (3.5±0.7 vs. 4.5±0.8, p<0.001) and of cyclosporin A (47.4±9.9 vs. 64.1±11.1, p<0.001). In addition, fewer patients in the intervention group received glucocorticoids 2 years after transplantation (20% vs. 47%, p=0.04). Caused by CD4 Tvis <2 cells/μl a total of 125 dose reductions of immunosuppressants (mainly based on ADV-Tvis) was performed in 28/31 children of the intervention group with a median of 4 Tvis-based dose reductions (range 0–10) per patient. Nearly half of the Tvis-based dose reductions were carried out in the first 6 months after transplantation. Only three Tvis-based dose increases were implemented in two children. Despite reduced immunosuppressive therapy, the number of biopsy-proven acute rejections did not increase in the intervention group but was even numerically lower than in the control group (p=0.11). Furthermore, the intervention group showed a numerically lower risk for EBV DNAemia (p=0.09). The numbers of DSAs and (serious) adverse events were comparable in both groups [34]. Consequently, the IVIST trial demonstrated that additional steering of immunosuppressive therapy by Tvis levels is safe and reduces exposure to immunosuppressive drugs with significantly lower trough levels but without increasing the risk of acute rejections. It is the first randomized controlled trial after pediatric kidney transplantation to prove that an effect-related drug monitoring by Tvis personalizes the pediatric post-transplant care and might serve as an additional marker that might complement—but obviously not replace—traditional trough level monitoring.

## Perspectives

In pediatric kidney transplantation, new diagnostic strategies, which mirror the individual cellular immune response such as Tvis, seem to be promising to decide on the necessity of antiviral medication and/or reduction of immunosuppressive therapy and thereby detect over-immunosuppression. The measurement of Tvis at the time of onset of viremia, challenging whether a therapeutic intervention should be performed, could become a part of routine practice. Prospective, interventional trials comparing standard of care with T cell-based steering of antiviral and immunosuppressive therapy in case of post-transplant viral infections are needed in order to confirm the usability of this antiviral management after pediatric transplantation. In addition to virus-specific immune response, the levels of Tvis represent the general cellular immune defense and thereby the intensity of immunosuppression after pediatric kidney transplantation serving as a marker to detect over-immunosuppression, avoid unnecessary exposure to immunosuppressive drugs, and reduce their undesirable side effects. Within a randomized controlled trial, the additional Tvis-based immune monitoring has proven to be safe and reduce immunosuppressive therapy after pediatric kidney transplantation, likely resulting in lower drug costs and thereby even in an economical benefit. Analysis of Tvis may become an important step towards the introduction of precision medicine in pediatric kidney transplantation to detect the individual intensity of immunosuppression, realize an effect-related drug monitoring, and target a tailor-made immunosuppressive therapy. With regard to existing data, Tvis seem to be a reliable indicator of over-immunosuppression but lesser effective to identify under-immunosuppression. Larger validation trials in separate patient cohorts are needed before this method can become standard of care. Cut-off values might have to be adopted in different patient populations as compared to the published levels.

Other immune markers, such as the TTV load, ImmuKnow®, QuantiFERON Monitor®, miRNA, or further cytokines and chemokines, may offer additional benefits for immune monitoring, but in contrast to Tvis-based management, none of these biomarkers has been evaluated within a randomized controlled trial after pediatric kidney transplantation. Further interventional trials are needed to investigate their potential for steering immunosuppression after solid organ transplantation. In the future, a panel using different parameters of cellular and humoral immune response in the urine and blood, including immunosuppressant trough levels but also Tvis, cytokines, miRNA, and others, could more accurately characterize the individual strength of the immune system of each kidney recipient to detect over- as well as under-immunosuppression. Artificial intelligence, that can use this panel to suggest an adjustment of immunosuppressive medication but which can also take into account all individual risk factors such as HLA-antigen mismatches, ischemia time, underlying disease, donor and recipient age, viral risk constellations, DSAs, prior rejection episodes, and virus DNAemia as well as the choice of immunosuppressive drugs and their specific side effects, might then become the next step in precision medicine for pediatric kidney recipients. This will herald a new era of personalized immunosuppressive therapy leading to better graft and patient survival in the future.

## Data Availability

Not applicable.

## Abbreviations

ADV: Adenovirus
ATP: Adenosine triphosphate
BKPyV: BK polyomavirus
CMV: Cytomegalovirus
DSA: Donor-specific antibody
EBV: Epstein-Barr virus
ELISA: Enzyme-linked immunosorbent assay
ELISpot: Enzyme-linked immunospot
FACS: Fluorescence-activated cell sorting
eGFR: Estimated glomerular filtration rate
HSV: Herpes simplex virus
IFNy: Interferon-gamma
MHC: Major histocompatibility complex
TTV: Torque teno virus
Tvis: Virus-specific T cells
