# Correction to: Novel ways to monitor immunosuppression in pediatric kidney transplant recipients—underlying concepts and emerging data

**DOI:** 10.1186/s40348-021-00124-w

**Published:** 2021-09-30

**Authors:** Thurid Ahlenstiel-Grunow, Lars Pape

**Affiliations:** Department of Pediatrics II, University Hospital of Essen, University of Essen-Duisburg, Hufelandstraße 55, 45147 Essen, Germany

**Correction to: Mol Cell Pediatr 8, 8 (2021)**

**https://doi.org/10.1186/s40348-021-00118-8**

Following publication of the original article [1], an error occurred in the authors’ competing interest statement during the typesetting of this article. The correct statement should read the following:

The authors declare that they have no competing interests concerning the content of this Mini Review. The authors have a bias towards their own published data.

We apologize for the inconvenience caused.

The original article has been corrected.
